# Evaluation of the Load-bearing Capacity of Fractured Incisal Edge of Maxillary Permanent Central Incisors restored with a Glass Fiber-reinforced Nanocomposite: An *in vitro* Study

**DOI:** 10.5005/jp-journals-10005-1278

**Published:** 2015-04-28

**Authors:** PS Praveen Kumar, KT Srilatha, B Nandlal, Kanika Singh Dhull

**Affiliations:** Associate Professor, Department of Dentistry, Mysore Medical College and Research Institute, Mysore, Karnataka, India; Professor, Department of Pedodontics and Preventive Dentistry, JSS Dental College, JSS University, Mysore, Karnataka, India; Professor, Department of Pedodontics and Preventive Dentistry, JSS Dental College, JSS University, Mysore, Karnataka, India; Reader, Department of Pedodontics and Preventive Dentistry, Kalinga Institute of Dental Sciences, KIIT University, Bhubaneswar Odisha, India

**Keywords:** Glass fiber-reinforced composite, Nanocomposite, Self-etching adhesive, Fracture resistance, Incisal edge fracture.

## Abstract

**Objectives:** The aim of this study was to evaluate and compare the load-bearing capacity of fractured incisal edge of maxillary permanent central incisors restored with a nanocomposite and a glass fiber-reinforced nanocomposite.

**Materials and methods:** Thirty-six extracted sound maxillary central incisors randomly divided in three groups were used for the present study. Group I (control) contained untreated teeth. Samples in experimental groups II and III were prepared by cutting the incisal (one-third) part of the crown horizontally and subjected to enamel preparations and restored with a nanocomposite and a glass fiber-reinforced nanocomposite respectively. All restored teeth were stored in distilled water at room temperature for 24 hours. Fracture resistance was evaluated as peak load at failure (Newton) for samples tested in a cantilever-bending test using Hounsfield universal testing machine. Failure modes were microscopically examined.

**Results:** Highest mean peak failure load (Newton) among experimental groups was observed in glass fiber-reinforced nano composite group (863.50 ± 76.12 N) followed by nanocomposite group (633.67 ± 40.14 N). One-way analysis of variance (ANOVA) revealed that the restoration technique significantly affected the load-bearing capacity (p < 0.001). Scheffe’s post-hoc comparison test (subset for α = 0.05) revealed that there was significant difference in the mean peak failure load values of nanocomposite and glass fiber-reinforced nanocomposite groups when considered together (p < 0.001). Experimental groups showed similar types of failure modes with majority occurring ascohesive and mixed type. Fifty-eight percent of the teeth in glass fiber-reinforced nanocomposite group fractured below the cementoenamel junction.

**Conclusion:** By using fiber-reinforced composite substructure under conventional composites in the repair of fractured incisors, the load-bearing capacity of the restored incisal edge could be substantially increased.

**How to cite this article:** Kumar PSP, Srilatha KT, Nandlal B, Dhull KS. Evaluation of the Load-bearing Capacity of Fractured Incisal Edge of Maxillary Permanent Central Incisors restored with a Glass Fiber-reinforced Nanocomposite: An *in vitro* Study. Int J Clin Pediatr Dent 2015;8(1):22-29.

## INTRODUCTION

Fracture of the crown of an anterior tooth is common and affects upto one-third of the patients in the pediatric and adolescent population.^1^ Dental trauma has both physical and psychological effects on a child by influencing both dental functions and appearance. A fractured anterior tooth in a young child confronts the dentist with a challenge for many reasons, such as the large pulp chamber that contraindicates certain restorations, incomplete root-end closure that may prevent root canal therapy and the impact of the psychological well being of the child and parents.^2^

Various techniques have been described to restore the fractured incisal edge to its original shape and color. Reattachment of the fractured incisal portion is a widely accepted and popularly established procedure. The esthetics of this method can be good, the problem of such restorations is their tendency to refracture or debond, most often due to a new trauma.^2^ Choices like a full-coverage restorations not only necessitates the removal of inordinate amounts of sound tooth structure, but also are time consuming and expensive.

Hence, the choice of treatment mostly narrows down to restorations with particulate filler composite (PFC) resins, owing to its esthetics and physical properties. However, contradictory results have been reported when PFCs are used for restoring anterior tooth fracture^1^ In fact, the number of these techniques attests to the difficulty in placement and frequent failure of this type of restoration. Shortcomings of such procedures are generally retention or esthetics.^3^

Fiber-reinforced composites (FRCs) have been tested as dental material and their use is growing in many dental applications, such as periodontal splints, endodontic posts, complete denture, removable and fixed partial denture, orthodontic appliances. Currently, the interest for using FRCs is rapidly growing and its use to reinforce long-term provisional restorations seems to have an acceptable success rate.

Studies have shown FRCs to have superior physical properties over PFCs. Although, a great deal is known about the properties of FRCs itself, less information is available on the properties of a material combination of FRC and PFC, when used as reinforcement of restorative composite resin.^1,4^

Thus, the purpose of this study was to evaluate and compare the load-bearing capacity of fractured incisal edges restored with a nanocomposite and a glass fiber-reinforced nanocomposite.

## MATERIALS AND METHODS

Thirty-six extracted intact human maxillary permanent central incisors were selected for this study. All teeth were extracted for therapeutic reasons in the Department of Oral and Maxillofacial Surgery at JSS Dental College and Hospital, Mysore. Institutional ethical committee approval was obtained prior to commencement of the study.

After removal of all adherent blood and soft tissue, they were stored in 0.5% chloramine-T bacteriostatic/ bacteriocidal solution for 1 week. Thereafter, they were stored in distilled water in a refrigerator at 4°C. In order to reduce deterioration, the storage medium was replaced perio dically.^5^ All teeth selected for testing were used within 1 month of procurement and were stored in distilled water at all the times when not in use.

Labial radiograph was taken and examined for crown, root and pulp space morphology to confirm similarity in configuration. Individual tooth intraoral periapical radiographs was taken to include/exclude the specimen for the study. Teeth were selected for similarity in size, shape and root anatomy. The cervicoincisal length on the labial surface and root length was measured using a digital Vernier caliper. This was done so that the teeth of similar dimensions could be evenly distributed between groups. Teeth within 1 mm difference of their mesio-distal incisal widths, with all or most of the incisal edge intact and with closed root apices were used for the study.

The test samples were prepared in the Department of Pedodontics and Preventive Dentistry, JSS Dental College Hospital, Mysore.

Using a mounting jig, all specimens were mounted in the same degree of angulations and vertical projection in aluminum casings with roots embedded in self-curing acrylic resin. Individual custom made strip crowns were fabricated on die models using positive pressure Biostar^®^ machine (Scheu Dental, Germany).

A standardized crown fracture was created using coordinate markings labially and lingually and joining them all around the tooth to form an imaginary horizontal fracture line, eliciting an Ellis C-II fracture. To ensure uniformity of crown restoration ratios, the incisal reduction were terminated 7 mm incisal to the labial cervical line. Experimental fractures were made with a diamond disk with water irrigation at a constant speed.^6^ The line joining the coordinates all around the tooth was narrowly preserved in order to control the sectioning process.

With specific standard diamond rotary instruments mounted in a high-speed air turbine handpiece, enamel preparations were carried out under distilled water spray.

**Fig. 1:**
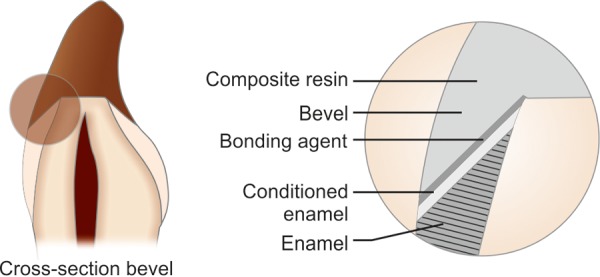
Schematic diagram of circumferential bevel (nanocomposite group)

*Nanocomposite group*: For samples in this group circumferential bevel was placed which extended 1 mm cervically from the fractured edge. The bevel included the entire thickness of enamel and extended from the dentinoenamel junction to the enamel surface (Fig. 1).

**Fig. 2:**
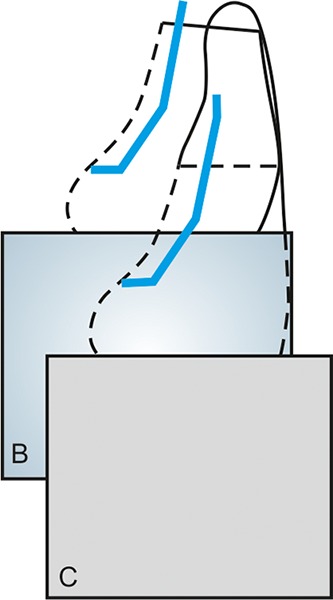
Schematic diagram of the palatal preparation (glass fiber-reinforced group)

**Fig. 3:**
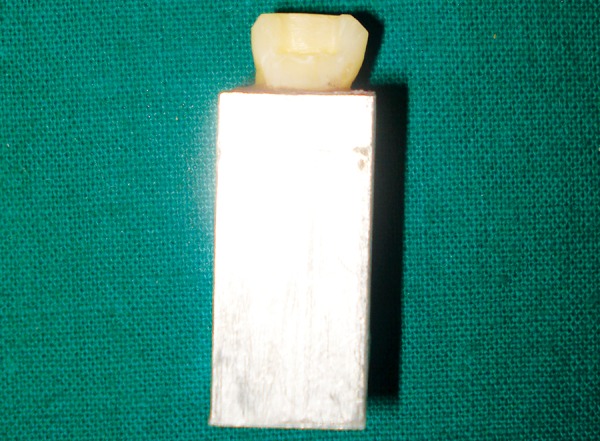
Palatal preparation (palatal view)

**Fig. 4:**
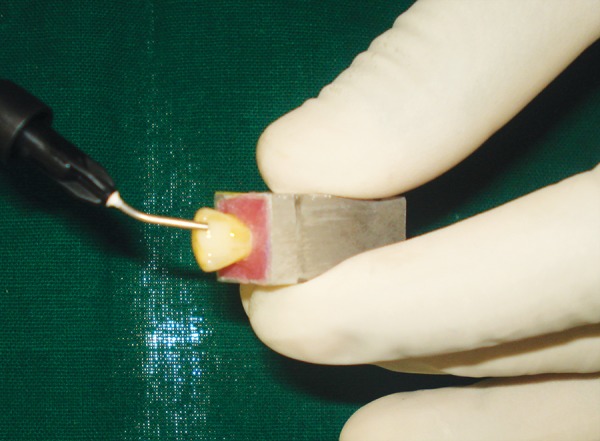
Flowable composite application

**Fig. 5:**
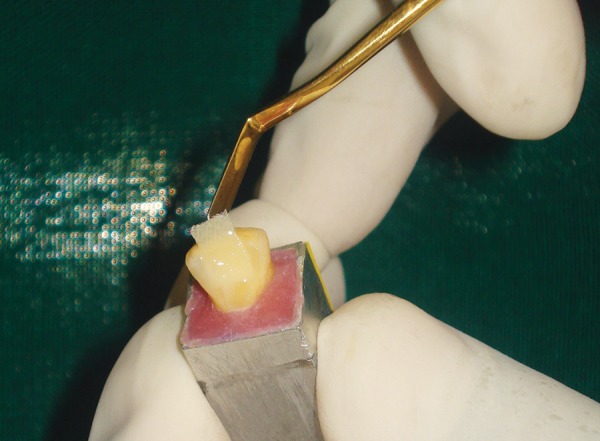
Placement of 4 × 4 mm glass fiber-reinforced composite

*Glass fiber-reinforced nanocomposite group*: A shallow palatal preparation (0.5 mm in depth, 5 mm in width and 3 mm in length) limited to enamel was prepared (Figs 2 and 3). A bevel was placed on the labial surface and extended palatally to include proximal margins of the palatal preparation. The bevel extended 1 mm cervically from the fractured edge and included the entire thickness of enamel and extended from dentino-enamel junction to the enamel surface. After the bonding technique, the glass fibers (Vectris Frame, Ivoclar Vivade nt AG, Fra nce) measuring 4 × 4 mm were cutusing a sharp scissor. A layer of flowable nanocomposite (Grandio Flow, VOCO, Germany) was placed in the palatal preparation (Fig. 4). The glass fibers were then gently carried and placed in the palatal preparation, such that 2 mm of glass fibers extended above the reduced incisal edge (Fig. 5). Using a soft-start polymerization curing unit (Translux^®^) glass fibers were light cured.

In both the groups, Xeno III (Dentsply DeTrey, Konstanz, Germany) single step self-etching dental adhesive system was applied on the prepared surface according to manufacturer’s instructions.

**Fig. 6:**
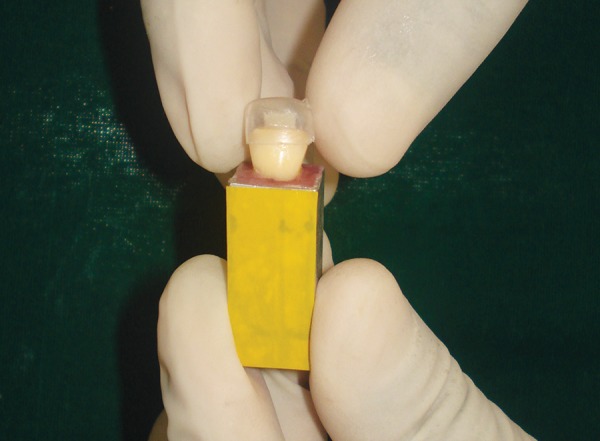
Custom made strip crown

**Fig. 7:**
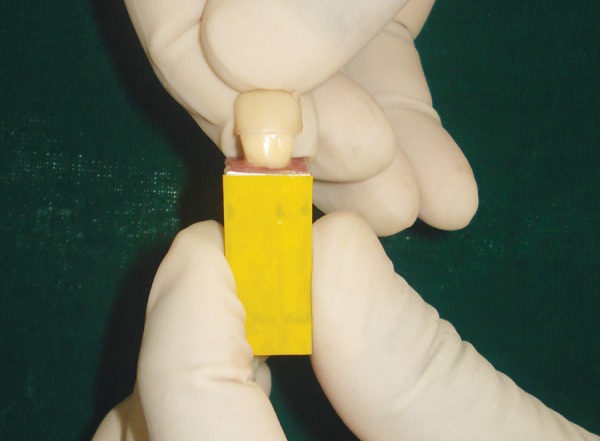
‘Bulk pack’ technique

**Fig. 8:**
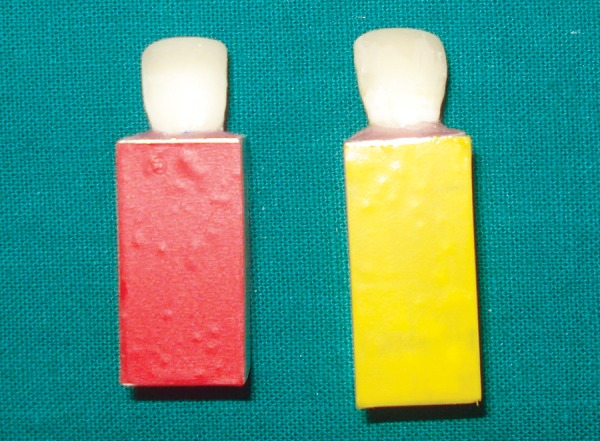
Completed restoration

Each tooth in both the groups was restored with use of custom strip crown formers (Fig. 6) in a ‘bulk pack’ technique using adequate amount of nanocomposite resin (Ceram-X Mono, Dentsply DeTrey, Konstanz, Germany) taken on a clean tefon-coated instrument and placed into the well of the crown former. Vent holes were placed at the mesial incisal corners to prevent entrapment of air and allow the escape of excess resin (Fig. 7). Using a soft-start polymerization curing unit (Translux^®^), composite resin was light cured through the strip crowns for one cycle of 40 seconds each from labial and lingual aspect. The tip of the curing light was placed in close approximation to all the samples. Strip crowns were gently removed after curing. Gross marginal excess resin was removed and finishing was done with composite finishing burs and fexible abrasive disks (Shofu, Japan) (Fig. 8). At this point, all crowns were remeasured to ensure that they were 11 mm long.

The rebuilt teeth were stored in distilled water at room temperature for 24 hours before testing.

The specimens were subjected to compressive loading in a Hounsfield universal testing machine (Hounsfield Equipment Ltd., UK) to determine the resistance to fracture. Before testing, blinding was done to eliminate the experimental bias.

**Fig. 9:**
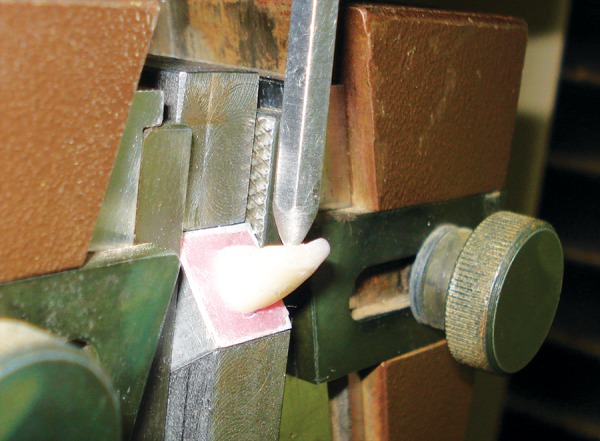
Specimen mounted at an angle of 130°

The compressive testing used a custom made stainless steel mounting block. The specimens were placed into the 50° angled face of the steel mounting block. The device was made in such a way that it allowed loading of tooth at an angle of 130° to its long axis (Fig. 9). This angle of loading was chosen to simulate a contact angle found in class I occlusion of maxillary and madibular anterior teeth.^7^ The direction, size and speed of the compressive head were selected so as to simulate as far as possible inter-incisal force that might be encountered in the mouth.

A loading force was applied along the predetermined standardized spot on lingual aspect of the specimen by a stainless steel 2 mm diameter, ball-ended compressive head at a constant crosshead speed of 1 mm per minute until the restoration was dislodged or fractured. The readings were noted to determine the peak force at failure in Newton (N). The results thus, obtained were subjected to statistical analysis. After testing all the specimens were examined microscopically to record and classify the mode of failure.

The actual mode of failure was recorded according to the following criteria:^2,8^

- Adhesive failure (A): Failure at tooth resin interface.
- Cohesive failure (C): Failure either within the body of the resin restoration or within the entire enamel thickness.
- Mixed (M): Partial failure of the resin restoration and partial adhesive failure at the interface.

## RESULTS

**Graph 1:**
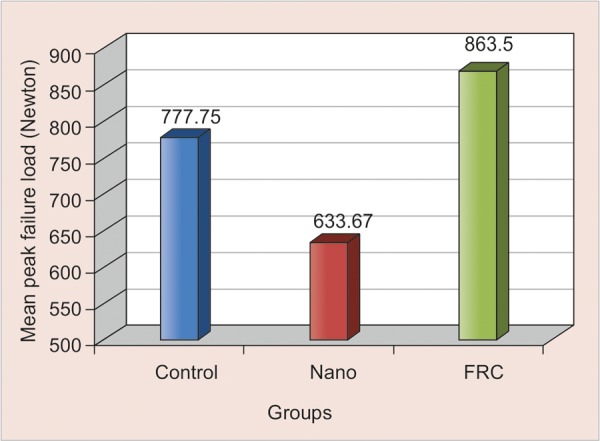
Mean peak failure load values of various groups

The mean peak failure load for control, nanocomposite and glass fiber-reinforced nanocomposite groups along with standard deviation values were 777.75 ± 82.22, 633.67 ± 40.14 and 863.50 ± 76.12 in Newton respectively (Table 1 and Graph 1). One-way A NOVA revealed asignificant difference in the mean peak failure load values of three different groups. F-value of 34.29 with 2 and 33 degrees of freedom is found to be highly significant (p < 0.001) (Table 2). The Scheffe’s post-hoc comparison test revealed that means of all the groups were statistically significant from each other mean peak failure load values, as indicated by the subsets formed (Table 3).

Independents samples t-test when applied between mean peak failure load values of control and nanocom-posite groups individually revealed ‘t’-value of 5.46 which was found to be highly significant (p < 0.001) (Table 4).

A significant difference was observed between mean peak failure load values of control group and glass fiber-reinforced nanocomposite group. Independents samples t-test revealed t-value of 2.66, which was found to be significant (p < 0.05) (Table 4).

Independents samples ‘t’-test when applied between mean peak failure load values of nanocomposite and glass fiber-reinforced nanocomposite groups individually revealed t-value of 9.26 which was found to be highly significant (p < 0.001) (Table 4).

**Graph 2:**
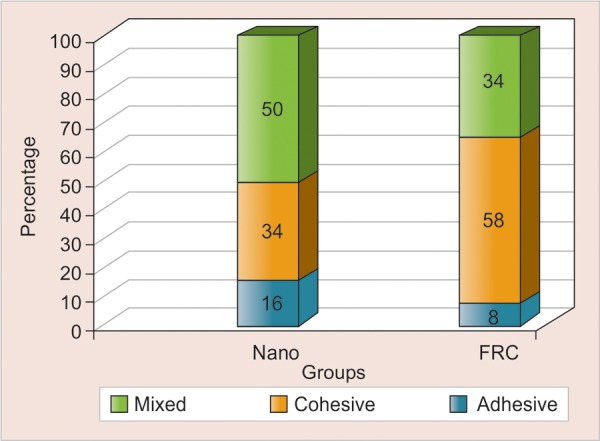
Percentage-wise distributions of failure modes in composite groups

Microscopic evaluation of the fracture site revealed the failure modes as shown in (Table 5 and Graph 2).

**Table 1:** **Table 1:** Descriptive statistics of peak failure load (Newton) for various groups

*Groups*		*Group description*		*n*		*Mean*		*SD*		*SE*	
I		Control (untreated)		12		777.75		82.22		23.74	
II		Nanocomposite		12		633.67		40.14		11.59	
III		Glass fiber-reinforced nanocomposite		12		863.50		76.12		21.98	
		Total		36		758.31		117.06			

**Table 2:** **Table 2:** Results of one-way ANOVA for peak failure load (Newton) for various groups

*Source of variation*		*Sum of squares*		*df*		*Mean square*		*F*		*Sig.*	
Between groups		323745.72		2		161872.861		34.283		0.000*** (p < 0.001)	
Within groups		155813.92		33		4721.634					
Total		479559.64		35							

df: degrees of freedom; F: Fishers value; p: Probability value;

***Highly significant

**Table 3:** **Table 3:** Results of Scheffe’s post-hoc test

				*Subset for α = 0.05*					
*Groups*		*n*		*1*		*2*		*3*	
Nanocomposite		12		633.67					
Control (untreated)		12				777.75			
Glass fiber-reinforced nanocomposite		12						863.50	

**Table 4:** **Table 4:** Summary of comparison between various groups using independent samples t-test

*Between groups*				*t-value*		*p-value*		*Inference*	
1. Control		- Nanocomposite		5.46		0.000		<0.001***	
2. Control		- Glass fiber-reinforced nanocomposite		2.66		0.015		<0.05*	
3. Nano composite		- Glass fiber-reinforced nanocomposite		9.26		0.000		<0.001***	

*Significant; ***Highly significant

## DISCUSSION

Numerous materials have been used traditionally to restore fractured anterior teeth. Selection of restorative materials that simulate the physical properties and other characteristics of natural teeth, in combination with newer adhesives provides framework for optimal development of an esthetic restoration that is durable. The introduction of acid-etch concept^9^ and composite resins as restorative material, has laid foundation for most of the present day techniques for the restoration of such teeth.

In the present study, attempt was made to simulate multifaceted oral conditions. The result of the present study takes into consideration some of the variables, amongst many others which may affect the fracture resistance of the composite resin restoration *in vivo*.

The methodology design employed in the present study was based on that of a previous study by Garoushi SK et al.^1^ It differed to their study in number of aspects. This study evaluated the fracture resistance of a nano-composite and a glass fiber-reinforced nanocomposite, used to restore fractured incisal edge. A single step self-etching dental adhesive was used.

Thirty-six extracted human maxillary permanent central incisors were used for the study which is the most ideal substrate for adhesion study like this, since the prevalence studies have revealed the extent of involvement of maxillary central incisor in uncomplicated fractures of anterior teeth resulting from direct trauma.^10-13^ Tooth specimens after procurement were handled and stored according to ISO specifications.^14^

Slow speed water-cooled diamond sectioning wheel blade was used to simulate standardized horizontal fracture.

Full thickness 1 mm bevel was placed on the teeth using standard rotary diamond burs. Currently, most clinicians prefer to use bevel because of conservative approach especially towards traumatized teeth and to gradient the color change from tooth matter to restoring material. It is also advocated for the ease of operation, conservative tooth preparation and reduced additional insult to recently traumatized teeth.^16^ Investigators have established that beveling the enamel surface would increase composite bonding strength to acid-etched enamel and decrease the chance of restoration failure.^15,17^

The bonding agent used was a single step self-etching adhesive (Xeno III). This is a newer generation bonding agent with minimal steps required for application and is less technique sensitive. They are extremely popular with Pedodontists as it reduces the operatory time and allows for the smoother treatment sessions, especially when dealing with anxious child patient who is distrustful of devices like three way syringe and suction hose. Thus, self etching adhesives have been introduced to simplify the bonding procedure.^18^

Ceram-X Mono (Dentsply DeTrey) used for the study is a light curable, radiopaque restorative material for anterior and posterior restorations of primary and permanent teeth. Based on proprietory nano-ceramic technology, Ceram-X Mono offers natural esthetics achieved by an easy procedure, superior handling characteristics and excellent durability.^19^

**Table 5:** **Table 5:** Percentage-wise distribution of failure modes at fracture site in composite groups

*Groups*		*Adhesive*		*Cohesive*		*Mixed*		*Total*	
Nanocomposite		2		4		6		12	
Percentage		16%		34%		50%			
Glass fiber-reinforced nanocomposite		1		7		4		12	
Percentage		8%		58%		34%			
Total		3		11		10		24	
Percentage		12%		46%		42%			

Vectris Frame (Ivoclar Vivadent) used in the study is a glass fiber-reinforced composite resin containing Bidirectional Woven E-glass (50% weight, 4-6 μm), pre-impregnated with Bis-GMA matrix (5% filler: SiO_2_).^20^

The ‘bulk pack technique’ of composite resin using preformed custom made strip crowns is a desired procedure in pediatric dental practice set-up to reduce the treatment time especially when dealing with patients with multiple carious lesions or an uncooperative child. Soft start polymerization was used to cure the composite resin using Translux^®^ energy (Heraeus Kulzer) in order to address the concern of stress buildup at the interface when using bulk pack technique, which could result in polymerization shrinkage of composite during curing.

Fracture mechanics approach has been used here to quantify the failure of composite resins, which were subjected to a dislodging force. Fracture resistance was evaluated as peak load at failure (Newton) for samples tested in a cantilever-bending test using Hounsfield universal testing machine.

Lingual side was chosen for the application of loading force to simulate oral conditions where trauma from biting of hard objects contributes maximum toward failure of such restorations. The point of load application was also very carefully controlled in order to maintain the same fulcrum distance for all the samples.^21^ In a simple mechanical model, if neutral axis were to remain located within the tooth, the cantilever bending would expose lingual surface to tension, whereas the labial surface would be exposed to compression. Therefore, the fracture will begin at the lingual side and on initiation of crack it would expose the labial surface to a complex bending load. Labial surface, which is exposed to these complex bending forces, was used for evaluating the type of failure at the fracture site.^22^

One of the assumptions necessary to make valid conclusions from the numerical data is that a normal distribution of data exists and that standard deviation among the groups is to a degree similar.

As observed from the data, the control group of untreated teeth showed substantially higher mean failure load values when compared to the nanocomposite group (Table 1 and Graph 1). It is evident that bonded teeth have significantly less capability than intact teeth to resist a dislodging force. Similar observations were made by the studies of Eid H^17^ and Eid H, White GE.^23^

Whereas glass fiber-reinforced nanocomposite group showed higher mean failure load values when compared to the control group. Due to insufficient amount of research conducted on this concept, a comparison in this regard was not possible. The possible rationalization for such an observation could be that by adding a FRC substructure under the nanocomposite, the load-bearing capacity of the material combination was increased as suggested by Garoushi et al^1^ and thus, have significantly more capability than intact teeth to resist a dislodging force.

As observed from the data, glass fiber-reinforced nanocomposite group comparatively showed substantially higher mean failure load values compared to the nanocomposite group (highly significant; p < 0.001). The findings of this study were in accordance to the findings of Garoushi SK et al,^1^ Belli S et al^24^ who observed higher mean failure values for FRC group than PFC group and concluded that use of FRC substructure under composite restorations significantly increased fracture strength. Similarly, studies by Vallitu PK et al,^25^ Garoushi S, Vallitu PK et al^4^ and Tezvergil A et al,^26^ observed a two to three times higher load bearing capacity of specimens with FRC substructures compared to that of PFC alone.

Higher (significant p < 0.001) values of glass fiber-reinforced nanocomposites compared to restoration with a nanocomposite alone, might be expected to have greater fracture resistance due to the fact that FRC is a group of materials having high toughness and strength that has been used in many applications in dentistry. In addition, the bond strength of chair-fabricated FRC to the dental tissue is as good as that of PFC.^26^ FRC combined with PFC is intended to provide better mechanical properties to the rebuilt incisal edge by distributing the forces to a wider surface area. This diminished stress at the interface and created a larger bonding area, which might have been beneficial under repeated loading.^1^

The complex compressive forces generated during the loading are likely to cause fracture through the restorative material before the shear components could operate at the interface between resin and enamel causing adhesive failures. In the present study, only 3/24 = 12% failures were recorded as adhesive (A) compared to 11/24 = 46% cohesive (C) and 10/24 = 42% as mixed (combination) (M) failure (Table 5 and Graph 2).

In glass fiber-reinforced nanocomposite group failures recorded were 1 (8%) adhesive, 7 (58%) cohesive (within tooth) and 4 (34%) mixed type respectively (Table 5 and Graph 2). Majority of the failures occurred below the cementoenamel junction as cohesive failure indicating high resistance to interfacial failure (adhesive) offered by FRC. This could be explained by the high strength of FRC, which exceeds the load-bearing capacity of the tooth, especially in teeth with thin roots.^1^

This failure pattern was similar to what observed from the study of Garoushi SK et al.^1^ Where in 50% of failures occurred below the cementoenamel junction as cohesive and 50% failures were categorized as failures within composite resin (combination failures were recorded as failures in resin).

It could be possibly inferred that material property of fracture resistance is best augmented when using glass fiber-reinforced nanocomposite as evident from high percentage of failures categorized as cohesive type, which could be explained by the high strength of FRC, which exceeds the load-bearing capacity of the tooth.

This test was carried out in *in vitro* conditions and the test was performed 24 hours after restoration. The thermal, chemical and physical stresses that the restoration could be subjected to over a longer period *in vivo* may affect the results; therefore, further investigation is necessary to predict the *in vivo* behavior of this restoration. Therefore, clinical evaluation of this technique over a period of time would be the true judgmental test.

## CONCLUSION

By using fiber-reinforced composite substructure under conventional composites in the repair of fractured incisors, the load-bearing capacity of the restored incisal edge was substantially increased. This might help to optimize properties of directly made composite restorations in anterior teeth and may offer an alternative toward overcoming some potential problems of composite restoration in high stress-bearing areas.

This technique has an excellent potential for greater clinical application, where incisally fractured teeth are restored with composite resin and reinforced with fibers, prov ides a higher load-bearing capacity than teeth restored with conventional technique.

## References

[1] Garoushi SK, Ballo AM, Lassila LV, Vallittu PK (2006). Fracture resistance of fragmented incisal edges restored with fiber-reinforced composite.. J Adhes Dent.

[2] Gandhi K, Nandlal B (2006). Effect of enamel preparations on fracture resistance of composite resin buildup of fractures involving dentine in anterior bovine teeth: an in vitro study.. J Ind Soc Pedod Prev Dent.

[3] Staffanou RS (1972). Restoration of fractured incisal angles.. JADA.

[4] Garoushi S, Lassila LV, Tezvergil A, Vallittu PK (2006). Load bearing capacity of fibre-reinforced and particulate filler composite resin combination.. J Dent.

[5] Titley KC, Chernecky R, Rossouw PE, Kulkarni GV (1998). The effect of various storage methods and media on shear bond strengths of dental composite resin to bovine dentine.. Arch Oral Biol.

[6] Hu YH, Pang C, Hsu CC, Lau YH (2003). Fracture resistance of endodontically restored with four post and core systems.. Quintessence Int.

[7] Tjan AHL, Dr Dent, Whang SB (1985). Resistence to root fracture of dowel channels with various thickness of buccal dentine walls.. J Prosthet Dent.

[8] Jordan RE, Suzuki M, Gwinnett AJ, Hunter JK (1977). Restoration of fractured and hypoplastic incisors by the acid etch technique: a three year report.. JADA.

[9] Buonocore MG (1955). A simple method of increasing the adhesion of acrylic filling materials to enamel surfaces.. J Dent Res.

[10] O’Neil DW, Clark MV, Lowe JW, Harrington MS (1989). Oral trauma in children : a hospital survey.. Oral Surg Oral Med Oral Pathol.

[11] Zerman N, Cavalleri G (1993). Traumatic injuries to permanent incisors.. Endod Dent Traumatol.

[12] Hamilton FA, Hill FJ, Holloway PJ (1997). An investigation of dento-alveolar trauma and its treatment in an adolescent population. Part 1: the prevalence and incidence of injuries and the extent and adequacy of treatment received.. Br Dent J.

[13] Bastone EB, Freer T, McNamara (2000). Epidemiology of dental trauma: a review of the literature.. Aust Dent J.

[14] De Munck J (2005). A critical review of the durability of adhesion to tooth tissue: Methods and results.. J Dent Res.

[15] Bagheri J, Denehy GE (1983). Effect of enamel bevel and restoration lengths on class IV acid-etch retained composite resin restoration.. JADA.

[16] Armstrong SR (1985). Restoration of class IV and VI defects in anterior teeth with an unfilled resin.. J Prosthet Dent.

[17] Eid H (2002). Retention of composite restorations in class IV preparations.. J Clin Pediatr Dent.

[18] Atash R, Van Den Abbeele A (2005). Bond strengths of eight contem-porary adhesives to enamel and to dentin: an in vitro study on bovine primary teeth.. Int J Pediatr Dent.

[19] Ceram X (2003). Scientific compendium..

[20] Martin AF (2000). et al. Fiber-reinforced composites in clinical dentistry..

[21] Van Noort R, Noroozi S, Howard IC, Cardew G (1989). A critique of bond strength measurements.. J Dent.

[22] Black JB, Retief DH, Lemons JE (1981). Effect of cavity design on retention of Class IV composite resin restorations.. JADA.

[23] Eid H, White GE (2003). Class IV preparations for fract ured anterior teeth restored with composite resin restorations.. J Clin Pediatr Dent.

[24] Belli S, Erdemir A, Ozcopur M, Eskitascioglu G (2005). The effect of fibre insertion on fracture resistance of root filled molar teeth with MOD preparations restored with composite.. Int Endod J.

[25] Garoushi SK, Lassila LV, Vallittu PK (2006). Fiber-reinforced composite substructure: load-bearing capacity of an onlay restoration.. Acta Odontol Scand.

[26] Tezvergil A, Lassila LV, Vallittu PK (2003). Strength of adhesive-bonded fiber-reinforced composites to enamel and dentin substrates.. J Adhes Dent.

